# Harley Street Associations

**Published:** 1920-12-11

**Authors:** 


### Harley Street Associations.

"A long dreary artery which gives the impression of having been cut out of cardboard" is Besant's far from flattering description of Harley Street; and one wonders whether in view of its present connection with physicians and surgeons, his selection of the term "artery" is deliberate.

Built about 1770, many of the houses contain fine architectural details, picturesque ceilings and staircases, and interesting ghosts may cluster o' nights around its massive fireplaces. In this street, in 1831, the unfortunate Lord and Lady Walsingham were burned to death in their beds; here, too, at No. 18, lived that pupil and imitator of Sir Joshua Reynolds, Sir William Beechey, portrait painter to Queen Charlotte.

No. 73 was the residence of Sir Charles Lyell, the eminent geologist and author, among other works, of the "Antiquity of Man," and after his death in 1875 the house was occupied by the Right Honourable (then plain Mr.) W. E. Gladstone.

No. 67 was at one time occupied by Allan Ramsay, appointed principal painter to George III.; Viscount Strangford, diplomatist and poet, also lived here; and, for a time, Lady Nelson, widow of the hero of Trafalgar.

Sir John Herschell, son of that Sir William Herschell, who discovered what he thought at first to be a comet and was, in fact, the planet now called Uranus, lived in Harley Street. Sir John, himself a fine astronomer and mathematician, was knighted at Queen Victoria's Coronation, and was for some years Master of the Mint.

One London topographer of the last century made an effort to establish a connection between Dean Swift and Harley Street and cites, as dated from that thoroughfare, one of the letters to "Stella" in which he refers to "The Mohawks," then infesting the ill-lighted streets of London. As the "Mohawks" flourished in 1712, the worthy Dean died in 1745, and Harley Street as we know it was built in 1770, we feel his zeal outran his discretion in this matter.

Dickens-lovers will hardly need to be reminded that the swindler "Mr. Merdle" in *Little Dorrit* had his residence in Harley Street.

